# Acute Effects of Gait Interventions on Tibial Loads During Running: A Systematic Review and Meta-analysis

**DOI:** 10.1007/s40279-022-01703-1

**Published:** 2022-06-16

**Authors:** Meghan Keast, Jason Bonacci, Aaron Fox

**Affiliations:** Centre for Sport Research, School of Exercise and Nutrition Sciences, Deakin University, 75 Pigdons Road, Waurn Ponds, VIC 3216 Australia

## Abstract

**Introduction:**

Changing running technique or equipment can alter tibial loads. The efficacy of interventions to modify tibial loads during running is yet to be synthesised and evaluated. This article reviewed the effect of running technique and footwear interventions on tibial loading during running.

**Methods:**

Electronic databases were searched using terms relevant to tibial load and running. Interventions were categorised according to their approach (i.e., footwear; barefoot running; speed; surface; overground versus treadmill; orthotics, insoles and taping; and technique); if necessary, further subgrouping was applied to these categories. Standardised mean differences (SMDs) with 95% confidence intervals (CIs) for changes in tibial loading were calculated and meta-analyses performed where possible.

**Results:**

Database searches yielded 1617 articles, with 36 meeting the inclusion criteria. Tibial loading increased with (1) barefoot running (SMD 1.16; 95% CI 0.50, 1.82); (2) minimalist shoe use by non-habitual users (SMD 0.89; 95% CI 0.40, 1.39); (3) motion control shoe use (SMD 0.46; 95% CI 0.07, 0.84); (4) increased stride length (SMD 0.86; 95% CI 0.18, 1.55); and (5) increased running speed (SMD 1.03; 95% CI 0.74, 1.32). Tibial loading decreased when (1) individuals ran on a treadmill versus overground (SMD − 0.83; 95% CI − 1.53, − 0.12); and (2) targeted biofeedback was used (SMD − 0.93; 95% CI − 1.46, − 0.41).

**Conclusions:**

Running barefoot, in motion control shoes or in unfamiliar minimalist shoes, and with an increased stride length increases tibial loads and may increase the risk of a tibial stress injury during periods of high training load. Adopting interventions such as running on a treadmill versus overground, and using targeted biofeedback during periods of high loads could reduce tibial stress injury.

## Key Points

**Table:** 

Running barefoot, in unfamiliar minimalist shoes, in motion control shoes, with increased stride length and increased speed increased tibial loading. Avoiding these conditions during training periods of high volume or when recovering from a tibial stress injury is recommended.
Running on a treadmill versus overground and the use of targeted biofeedback reduced tibial loading. These strategies could be adopted to reduce tibial loads in training or rehabilitation.

## Introduction

Two-thirds of people who meet or exceed physical activity guidelines use running as a mode of exercise [1]. Those who engage in high volumes of running are at an elevated risk of sustaining lower limb bone stress injuries [2, 3]. Stress injuries to the tibia are the fifth (tibial stress syndrome) and ninth (tibial stress fractures) most common running injury; combined, they account for approximately 1 in 10 running injuries [4]. Management and rehabilitation of a tibial stress injury involves a period of rest from high impact exercise and/or full immobilisation, followed by a slow integration back into exercise and sport [5, 6]. The average passive rest period is 8.3 weeks [5], with return to normal activities taking up to 17 weeks depending on severity [7]. These periods away from training and exercise can cause significant reductions in aerobic, anaerobic and muscular fitness in as little as 4 weeks [8]. Prevention of tibial stress injuries is important to avoid the induced inactivity that could impact athlete performance and potential negative health outcomes.

Tibial stress injuries result, in part, from the mechanical fatigue of bone [9]. Cyclic impact loading over time generates minuscule bone cracks (i.e., microdamage) that have the potential to degrade bone material properties [10]. The amount of microdamage can be affected by several variables, including volume, magnitude, and frequency of loads. In healthy individuals, there is a balance between the reabsorption and formation of bone, and strain induced microdamage is removed successfully [10]. However, bone can go into a state of accelerated remodelling if there is inadequate time for successful removal of microdamage. Repeated strain of a high magnitude, without adequate rest or lower magnitude strains coupled with a large volume (i.e., high number of running cycles) or high rate of loading can cause accelerated remodelling to occur [11, 12]. Bone reabsorption will begin to outpace the formation of bone, and microdamage will begin to accumulate. If adequate rest is not introduced in either scenario, a tibial stress fracture can occur [11, 12]. Tibial stress injuries are multifactorial in nature and can be affected by both non-modifiable (i.e., sex, bone density and skeletal alignment) and modifiable risk factors (i.e., training volume and intensity, running biomechanics, equipment and surfaces) [13–15]. Changing running technique and biomechanics is one way to alter the load and stress placed on bone [16]. Gait retraining has been used as a tool to modify tibial loads [16–20] through changing speed [19, 20], stride length [21, 22] and cadence [18], step width [16] and foot strike pattern [17, 18]. Foot orthoses and footwear have also been used to modify tibial load [23–26]. The efficacy of these interventions to change tibial loading is yet to be synthesised and compared, which may help identify the most effective means for reducing tibial loads. The purpose of this review is to synthesise and evaluate the effect of technique and footwear interventions on tibial loads via meta-analyses.

## Methods

This systematic review and meta-analysis was conducted in accordance with the Preferred Reporting Items for Systematic Reviews and Meta-Analyses (PRISMA) guidelines [27]. The review protocol was registered on the Open Science Framework (21 September 2020; https://osf.io/vm7fk).

### Literature Search Strategy

The Academic Search Complete, SPORTDiscus, Medline compete and CINAHL complete databases were searched, with the last search being run on 12 July 2021. Two searches were run, the first used the key terms and Boolean operators: (tibia*) AND (stress* OR strain*) AND (injur* OR “injury prevention” OR “injury reduction”) AND (intervention OR train* OR program OR strateg*), and the second search aimed at targeting specific intervention types and was run using the terms and Boolean operators: (tibia*) AND (stress* OR strain* OR strain) AND (injur* OR “injury prevention” OR “injury reduction”) AND (step* OR stride* OR shoe* OR orthotic* OR speed OR velocity). No additional filters or search limitations were used. Following these database searches, all titles and abstracts were extracted, and two reviewers (MK, AF) independently reviewed these for relevance. Any conflicts were resolved through consensus. Where consensus could not be reached, a third reviewer (JB) was consulted. Full-text articles were obtained for all articles deemed relevant. The full-text articles were also reviewed independently by the same two reviewers. Articles were assessed for relevance against a set of inclusion and exclusion criteria (see Sect. 2.2, Eligibility). Those articles that met the criteria were included for data extraction. Reference lists of included studies were also reviewed manually to identify any remaining publications. Any titles found to be relevant in the reference lists underwent the same abstract and full-text screening process.

### Eligibility

Studies were included if they (1) examined individuals between the ages of 18 and 45 years; and (2) evaluated the immediate effect of a gait retraining or footwear intervention on a relevant measure of tibial loads during running [28–32]. Gait retraining interventions were considered as those that aimed to modify the participants’ running technique by using targeted technique changes, footwear, surfaces etc. Relevant measures of tibial loading included were (1) stress or strain measures obtained from strain gauges embedded in the tibia, or finite element models; (2) impulse, peak tibial acceleration or impact forces from inertial sensors or accelerometers placed on the tibia; and (3) joint reaction or contact forces at the ankle estimated from musculoskeletal models. Studies that only investigated ground reaction force measures to infer tibial loading were excluded due to ground reaction forces alone not being well correlated with bone strain measures [33]. Recent studies have also implied that wearable sensors such as accelerometers may have the same poor correlations with tibial loading measurements as that of ground reaction forces, particularly at the bone level [33, 34]. While we acknowledge these limitations of tibial acceleration measures, particularly on inclined surfaces, they have been included in this review due to associations with tibial stress injury [35]. Studies were excluded from the review if (1) animal or cadaveric models were used; (2) data from a baseline or control condition to compare the intervention effect were not available; and (3) the full text was not accessible.

### Risk of Bias

Risk of bias of the included studies was assessed using a modified version of the Cochrane Collaboration’s tool for assessing risk of bias in randomised trials [36]. The modified version removed the performance bias criterion as, in most cases, it was considered impossible to blind participants to the interventions used. The authors deemed it important that participants had familiarisation with the interventions, and therefore a familiarisation bias was added. Lastly a statistical bias criterion was added; studies needed to use an appropriate statistical method for paired data and provide an estimate of variability (i.e., standard deviation, standard error) to be evaluated as low risk of bias.

### Data Extraction

Data were extracted from the included articles by a single reviewer (MK). The following data were extracted from the included studies: (1) participant characteristics (i.e. number of participants, male/female ratio, age, height, weight, running kilometres/mileage per week and running experience where available); (2) details of the gait retraining technique used; (3) how running technique was assessed (i.e. overground vs. treadmill, running distance and speed); and (4) the tibial load assessment method (i.e. strain gauge, finite element analysis, accelerometer, musculoskeletal modelling) and measure (i.e. stress, strain, tibial acceleration or impact forces, joint reaction or contact forces) used. Corresponding authors were contacted where data could not be extracted from the full text alone. If no response was provided and data could not be extracted, the study was excluded from further analysis.

### Data and Statistical Analysis

Studies were categorised based on the gait retraining intervention. Based on the set of included studies, each intervention and corresponding data were separated into seven categories: (1) barefoot (i.e. a change from shod to barefoot running); (2) footwear (i.e. modification or change in footwear); (3) speed (i.e. modification to running speed); (4) surface (i.e. modification in running surface); (5) overground versus treadmill (i.e. comparison of overground to treadmill running); (6) orthotics, insoles and taping (i.e., interventions that included the use of orthotics, cushioned insoles or taping/bracing methods); and (7) technique (i.e., biomechanical modifications to running technique). These were separated into further subcategories where appropriate. Footwear interventions were separated into five subcategories: (1) high-cut shoes; (2) shoes with increased cushioning; (3) minimalist shoes (in habitual wearers); (4) minimalist shoes (in non-habitual wearers); and (5) motion control shoes. Orthotics, insoles, and taping interventions were separated into a further five subcategories: (1) cushioned insoles; (2) rigid orthotics; (3) semi-rigid orthotics; (4) soft orthotics; and (5) taping and bracing. Surface interventions were separated into four subcategories: (1) grass; (2) normal versus a padded treadmill; (3) synthetic surfaces; and (4) woodchips. Finally, technique interventions were separated into nine subcategories, (1) anterior trunk lean; (2) increased cadence; (3) forefoot strike; (4) real-time biofeedback; (5) grounded running; (6) increased stride length; (7) decreased stride length; (8) increased step width; and (9) decreased step width. Standardised mean differences (SMDs) with 95% confidence intervals (CIs) were calculated between the baseline and intervention condition(s) for all studies, and meta-analyses were performed where possible to identify pooled effects.

Random-effects meta-analyses were conducted (where possible) using STATA version 16 (StataCorp LLC, College Station, TX, USA). Categories that contained a single intervention were unable to be pooled for meta-analyses. The meta-analyses used mean and standard deviation to calculate SMDs (Hedges’ *g*) with 95% CIs. Due to several studies having small sample sizes (i.e., < 20), Hedges’ *g* [37] was opted for over Cohen’s *d* [38]. Effect sizes were interpreted as trivial (< 0.20), small (0.20–0.59), moderate (0.60–1.19), large (1.20–1.99), and very large (≥ 2.00) [39]. Heterogeneity of studies was assessed using the Higgins *I*^2^ statistic. *I*^2^ percentages were interpreted as small (> 25%), medium (> 50%) or high (> 75%) levels of heterogeneity between studies [40].

The certainty of pooled evidence was evaluated using the Grading of Recommendations Assessment, Development and Evaluation (GRADE) system [41]. Randomised controlled trials were rated as high-grade evidence and observational studies started as low-grade evidence. The overall quality was then downgraded one level to moderate, low and/or very low dependent on the following criteria: imprecision (total sample size < 400 participants), inconsistency (high statistical heterogeneity > 50%), and risk of bias (more than 50% of studies having one or more risk-of-bias item criterion considered high risk).

## Results

### Literature Search Results

**Fig. 1:**
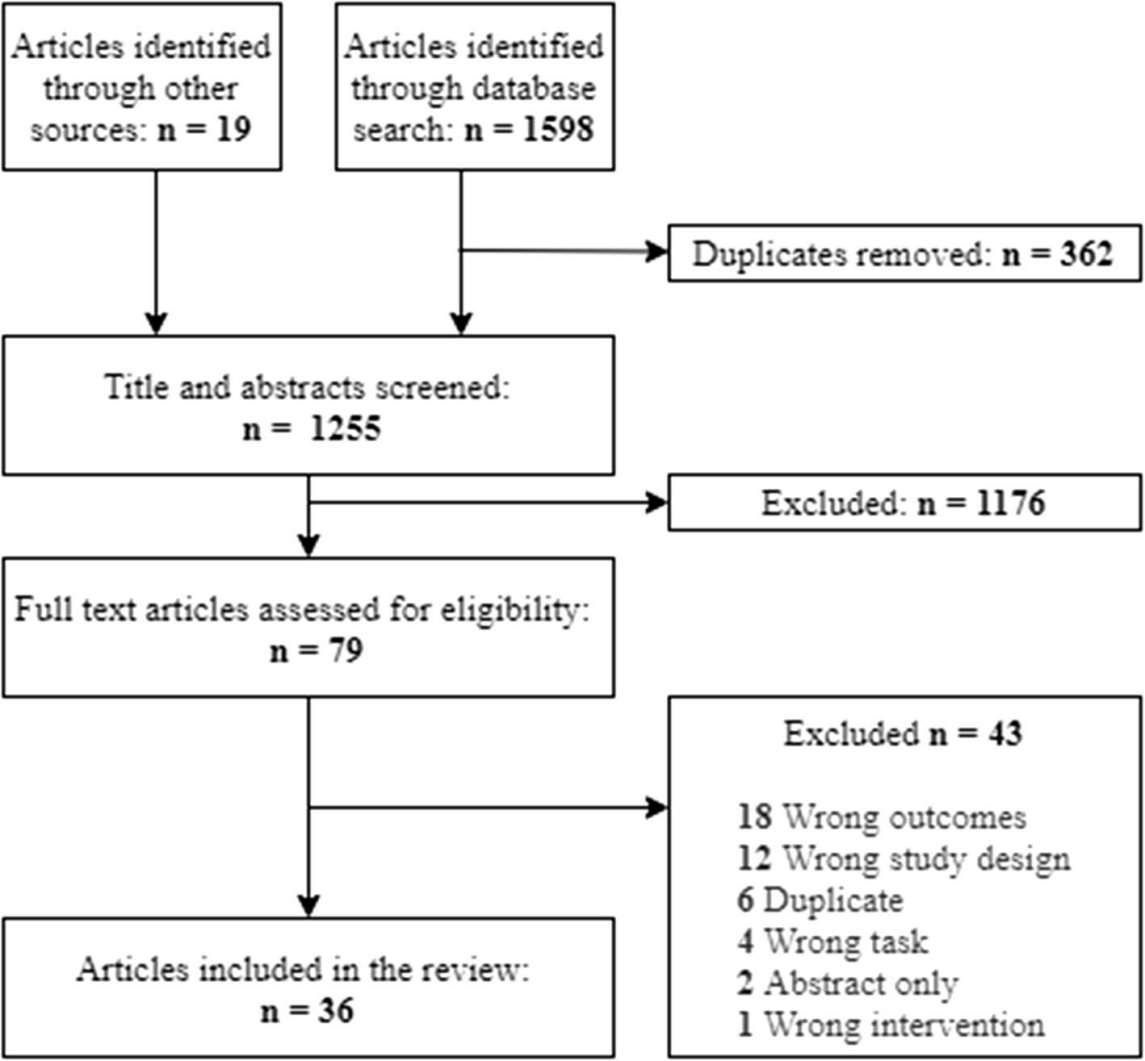
Study selection process

The electronic database and reference list searches yielded a total of 1617 articles for screening, of which 362 were found to be duplicates and were removed prior to title and abstract screening (Fig. 1). Title and abstract screening found 79 potentially relevant articles, of which 43 were removed during full-text screening. The final number of studies included in the review was 36.

### Study Characteristics

All studies were a crossover design, with 19 randomising the order of conditions. The included articles were published between 1996 and 2021 and included 677 participants (of those, 420 were known to be male, 186 were female, and 71 were not specified). Eighty-two interventions were extracted from the 36 included articles (i.e., certain studies included multiple interventions). Of these interventions, four were categorised as barefoot; 11 as footwear; 11 as orthotic, insole and taping; three as overground versus treadmill; 21 as speed; 10 as surface; and 20 as technique-based interventions. Two surface and one speed intervention extracted from the same study [42], and one speed intervention from a separate study [43], were not included in the meta-analysis due to an insufficient number of participants (*n* = 1). The methods used to evaluate tibial loads included accelerometers placed on the tibia (*n* = 27, 75%), musculoskeletal models (*n* = 3, 8.33%), finite element models (*n* = 2, 5.56%) and tibia-embedded strain/stress gauges (*n* = 4, 11.11%). Detailed study characteristics for each intervention category are reported in Tables 1, 2, 3, 4, 5, 6 and 7.

**Table 1:** Participant and intervention characteristics of studies included in the barefoot category

Study	*N*	Age (years)	Height (cm)	Mass (kg)	Population activity (km run per week)	Subcategory (if applicable)	Control condition	Intervention condition	Tibial load measure
Lucas-Cuevas et al. [44]	22 *NS*	28.4 ± 5.8	173.6 ± 5.9	68.5 ± 6.6	Recreational runners (38.6 ± 15.4)	NA	Conventional running shoe	Non-habitual participants running barefoot without prior training	Peak tibial acceleration (g) via accelerometer
Olin and Gutierrez [45]	6 M,12 F	32.2 ± 7.9	172 ± 10	68.6 ± 11.9	Recreational runners (20.9 ± 6.0)	NA	Participant’s normal running shoe	Non-habitual participants running barefoot without prior training	Peak tibial shock (g) via accelerometer
Sinclair et al. [46]	10 M, 0 F	20.42 ± 3.55	178.75 ± 5.81	76.58 ± 6.52	Recreational runners (≥ 35)	NA	Conventional running shoe	Non-habitual participants running barefoot without prior training	Peak tibial acceleration (g) via accelerometer
Sinclair et al. [23]	10 M, 0 F	24.3 ± 1.1	178.1 ± 5.2	76.79 ± 8.96	Experienced runners (≥ 30)	NA	Conventional running shoe	Non-habitual participants running barefoot without prior training	Peak tibial acceleration (g) via accelerometer

*NS* not specified, *NA* not applicable, *M* males, *F* females

**Table 2:** Participant and intervention characteristics of studies included in the footwear category

Study	*N*	Age (years)	Height (cm)	Mass (kg)	Population activity (km run per week)	Subcategory (if applicable)	Control condition	Intervention condition	Tibial load measure
Sinclair et al. [47]	12 M, 0 F	22.47 ± 1.13	1.77 ± 0.08	80.32 ± 6.33	Collegiate American football players (NS)	High-cut footwear	Low-cut football cleat (Nike Vapor pro low TD)	High-cut football cleat (Nike Lunar code pro)	Peak tibial acceleration (g) via accelerometer
Lam et al. [48]	18 M, 0 F	25 ± 2.3	179 ± 4.6	74.4 ± 6.5	Collegiate basketball players (NS)	Increased cushioning	Conventional running shoe	Running shoe with medium cushioning	Tibial shock (g) via an accelerometer
Lam et al. [48]	18 M, 0 F	25 ± 2.3	179 ± 4.6	74.4 ± 6.5	Collegiate basketball players (NS)	Increased cushioning	Conventional running shoe	Running shoe with high cushioning	Tibial shock (g) via an accelerometer
Sinclair et al. [49]	20 M, 0 F	30.59 ± 4.97	173 ± 4	70.25 ± 6.43	Recreational runners (≥ 35)	Minimalist footwear (H)	Conventional running shoe (New Balance 1260 V2)	Habitual minimalist shoe wearers, wearing a minimalist shoe (Vibram Five Fingers, ELX)	Peak tibial acceleration (g) via accelerometer
Izquierdo-Renau et al. [50]	17 M, 0 F	37.94 ± 9.64	177 ± 7	73.87 ± 8.97	Experienced runners (≥ 20)	Minimalist footwear (H)	Conventional running shoe (participants’ preferred shoe)	Habitual minimalist shoe wearers, wearing a minimalist shoe (participants’ preferred shoe)	Peak tibial acceleration (g)
Sinclair et al. [49]	20 M, 0 F	30.59 ± 4.97	173 ± 4	70.25 ± 6.43	Recreational runners (≥ 35)	Minimalist footwear (NH)	Conventional running shoe (New Balance 1260 V2)	Non-habitual minimalist shoe wearers wearing a minimalist shoe (Vibram Five Fingers, ELX)	Peak tibial acceleration (g) via accelerometer
Sinclair et al. [23]	10 M, 0 F	24.3 ± 1.1	178.1 ± 5.2	76.79 ± 8.96	Experienced runners (≥ 30)	Minimalist footwear (NH)	Conventional running shoe (Saucony Pro Guide 2)	Barefoot inspired/minimalist shoe (Nike Free 3.0)	Peak tibial acceleration (g) via accelerometer
Sinclair et al. [46]	10 M, 0 F	20.42 ± 3.55	178.75 ± 5.81	76.58 ± 6.52	Recreational runners (≥ 35)	Minimalist footwear (NH)	Conventional running shoe (Saucony Pro Guide 2)	Barefoot inspired/minimalist shoe (Vibram Five Fingers, ELX)	Peak tibial acceleration (g) via accelerometer
Butler et al. [24]	19 M, 21 F	22.5 ± 4.5	172 ± 9	68.91 ± 9.09	Recreational runners (≥ 16.1)	Motion control footwear	High-arched participants wearing conventional running shoe (New Balance 1022NC)	High-arched participants wearing a motion control shoe (New Balance 1122MC)	Peak positive tibial acceleration (g) via an accelerometer
Butler et al. [24]	19 M, 21 F	22.5 ± 4.5	172 ± 9	68.91 ± 9.09	Recreational runners (≥ 16.1)	Motion control footwear	Low-arched participants wearing conventional running shoe (New Balance 1022NC)	Low-arched participants wearing a motion control shoe (New Balance 1122MC)	Peak positive tibial acceleration (g) via an accelerometer
Butler et al. [51]	24 NS	21.4 ± 3.1	172 ± 9	69.20 ± 6.58	Recreational runners (≥ 16.1)	Motion control footwear	Low-arched participants wearing conventional running shoe (New Balance 1022NC)	Low-arched participants wearing a motion control shoe (New Balance 1122MC)	Peak positive tibial acceleration (g) via an accelerometer

*NS* not specified, *H* habitual *NH* non habitual, *M* males, *F* females

**Table 3:** Participant and intervention characteristics of studies included in the orthotics, insoles, and taping category

Study	*N*	Age (years)	Height (cm)	Mass (kg)	Population activity (km run per week)	Subcategory (if applicable)	Control condition	Intervention condition	Tibial load measure
O’Leary et al. [53]	7 M, 9 F	20–36	173 ± 9	68.4 ± 12	Recreational runners (NS)	Cushioned insoles	Conventional running shoe (New Balance M635 or W630)	Prefabricated cushioned insoles (Sorboair) inserted into the conventional running shoe (New Balance M635 or W630)	Peak tibial acceleration (g) via accelerometer
Lucas-Cuevas et al. [52]	20 M, 18 F	29.8 ± 5.3	170.3 ± 11.4	65.4 ± 10.1	Recreational runners (36.5 ± 7.2)	Cushioned insoles	The original sock liner of the participants’ preferred running shoe	Prefabricated cushioned insoles (NS) inserted into the participants’ preferred running shoe	Peak tibial acceleration (g) via accelerometer
Lucas-Cuevas et al. [52]	20 M, 18 F	29.8 ± 5.3	170.3 ± 11.4	65.4 ± 10.1	Recreational runners (36.5 ± 7.2)	Cushioned insoles	The original sock liner of the participants’ preferred running shoe	Custom-made cushioned insoles inserted into the participants’ preferred running shoe	Peak tibial acceleration (g) via accelerometer
Laughton et al. [26]	15 NS	22.46 ± 4	169.75 ± 6.07	66.41 ± 8.58	Runners (NS)	Rigid orthotics	Conventional running shoe (Nike Air Pegasus)	Conventional running shoe (Nike Air Pegasus) with subject-specific rigid orthotics fabricated using suborthelene	Peak positive tibial acceleration (g) via an accelerometer
Butler et al. [25]	15 M, 0 F	18–45	NS	NS	Recreational runners (≥ 10)	Rigid orthotics	Conventional running shoe (Nike Air Pegasus)	Conventional running shoe (Nike Air Pegasus) with subject-specific rigid orthotics fabricated using suborthelene	Peak positive tibial acceleration (g) via an accelerometer
Ekenman et al. [54]	9 NS	32.4 ± NS	183.9 ± NS	82.2 ± NS	Physically active—Swedish police force (NS)	Semi-rigid orthotics	Conventional running shoe (Nike Air Max)	Conventional running shoe (Nike Air Max) with semi-rigid orthotics	Peak-to = peak strain via two instrumented staples
Ekenman et al. [54]	9 NS	32.4 ± NS	183.9 ± NS	82.2 ± NS	Physically active—Swedish police force (NS)	Soft orthotics	Conventional running shoe (Nike Air Max)	Conventional running shoe (Nike Air Max) with soft orthotics	Peak-to-peak strain via two instrumented staples
Butler et al. [25]	15 M, 0 F	18–45	NS	NS	Recreational runners (≥ 10)	Soft orthotics	Conventional running shoe (Nike Air Pegasus)	Conventional running shoe (Nike Air Pegasus) with subject-specific soft orthotics fabricated using EVA Foam	Peak positive tibial acceleration (g) via an accelerometer
Kersting et al. [55]	10 M, 0 F	28.9 ± 5	178.1 ± 2.7	76.3 ± 6.3	Experienced runners (NS)	Tape and bracing	Conventional running shoe (Asics Gel 121)	Conventional running shoe (Asics Gel 121) with ankle taping using 4 cm medical tape	Peak tibial acceleration (g) via an accelerometer
Kersting et al. [55]	10 M, 0 F	28.9 ± 5	178.1 ± 2.7	76.3 ± 6.3	Experienced runners (NS)	Tape and bracing	Conventional running shoe (Asics Gel 121)	Conventional running shoe (Asics Gel 121) with an ankle cast/brace (Air-stirrup, Aircast)	Peak tibial acceleration (g) via an accelerometer
Kersting et al. [55]	10 M, 0 F	28.9 ± 5	178.1 ± 2.7	76.3 ± 6.3	Experienced runners (NS)	Tape and bracing	Conventional running shoe (Asics Gel 121)	Conventional running shoe (Asics Gel 121) with ankle taping using 4 cm medical tape and an ankle cast/brace (Air-stirrup, Aircast)	Peak tibial acceleration (g) via an accelerometer

*NS* not specified, *M* males, *F* females

**Table 4:** Participant and intervention characteristics of studies included in the overground versus treadmill category

Study	*N*	Age (years)	Height (cm)	Mass (kg)	Population activity (km run per week)	Subcategory (if applicable)	Control condition	Intervention condition	Tibial load measure
Milgrom et al. [57]	2 M, 1 F	39.33 ± 15.56	NS	73.33 ± 11.6	2 recreational runners (≥ 10) 1 tennis player (NS)	NA	Concrete	Standard treadmill	Micro strain via an instrumented staple inserted on the medial aspect of the mid-diaphysis of the tibia
Milner et al. [58]	9 M, 10 F	31 ± 6	170 ± 8	68.6 ± 11.6	Healthy runners (≥ 16.1)	NA	Concrete	Standard treadmill	Peak tibial acceleration (g) via accelerometer
Fu et al. [56]	13 M, 0 F	23.7 ± 1.2	173.7 ± 5.7	65.7 ± 5.2	Recreational runners (20.4 ± 5.2)	NA	Concrete	Standard treadmill	Peak positive tibial acceleration (g) via an accelerometer placed on the tibial tuberosity

*NS* not specified, *NA* not applicable, *M* males, *F* females

**Table 5:** Participant and intervention characteristics of studies included in the speed category

Study	*N*	Age (years)	Height (cm)	Mass (kg)	Population activity (km run per week)	Subcategory (if applicable)	Control condition	Intervention condition	Tibial load measure
Mercer et al. [62]	8 M, 0 F	25 ± 4.6	179 ± 6	80 ± 8.9	Physically active (NS)	NA	3.2 m/s	3.8 m/s	Peak tibial acceleration (g) via accelerometer
Meardon et al. [63]	0 M, 20 F	24.65 (23.16–26.14)	167.97 (165.18–171.76)	58.22 (53.99–62.45)	Recreational runners (30.98 [23.72–38.24])	NA	90% of preferred running speed	Preferred running speed	Anterior posterior bending moment of the tibia (Nm)
Meardon et al. [63]	20 M, 0 F	24.95 (22.87–27.03)	181.30 (178.44–184.17)	80.08 (76.13–84.02)	Recreational runners (24.34 [17.90–30.78])	NA	90% of preferred running speed	Preferred running speed	Anterior posterior bending moment of the tibia (Nm)
Meardon et al. [63]	0 M, 20 F	24.65 (23.16–26.14)	167.97 (165.18–171.76)	58.22 (53.99–62.45)	Recreational runners (30.98 [23.72–38.24])	NA	90% of preferred running speed	110% of preferred running speed	Anterior posterior bending moment of the tibia (Nm)
Boey et al. [59]	18 M, 17 F	23.3 ± 3.33	175.77 ± 8.71	64.74 ± 7.69	Untrained runners (< 2) Recreational runners (18 ± 7) Well-trained runners (74 ± 19)	NA	3.06 m/s	3.67 m/s	Positive vertical acceleration peaks (g) via accelerometer
Sheerin et al. [31]	65 M, 20 F	39.6 ± 9.0	176 ± 9	73.9 ± 11	Runners (45.0 ± 23.2)	NA	2.7 m/s	3.0 m/s	Peak tibial acceleration (g) via accelerometer
Hunter et al. [20]	14 M, 29 F	24 ± 6	168 ± 10	63.12 ± 9.61	Recreational runners (40 [range 10–113])	NA	2.70 m/s	3.27 m/s	Tibial load (bw) estimated using musculoskeletal modelling
Meardon et al. [63]	20 M, 0 F	24.95 (22.87–27.03)	181.30 (178.44–184.17)	80.08 (76.13–84.02)	Recreational runners (24.34 [17.90–30.78])	NA	90% of preferred running speed	110% of preferred running speed	Anterior posterior bending moment of the tibia (Nm)
Mercer et al. [62]	8 M, 0 F	25 ± 4.6	179 ± 6	80 ± 8.9	Physically active (NS)	NA	3.2 m/s	4.5 m/s	Peak tibial acceleration (g) via accelerometer
Sheerin et al. [31]	65 M, 20 F	39.6 ± 9.0	176 ± 9	73.9 ± 11	Runners (45.0 ± 23.2)	NA	2.7 m/s	3.3 m/s	Peak tibial acceleration (g) via accelerometer
Hunter et al. [20]	14 M, 29 F	24 ± 6	168 ± 10	63.12 ± 9.61	Recreational runners (40 [range: 10–113])	NA	2.70 m/s	4.08 m/s	Tibial load (bw) estimated using musculoskeletal modelling
Mercer et al. [62]	8 M, 0 F	25 ± 4.6	179 ± 6	80 ± 8.9	Physically active (NS)	NA	3.2 m/s	5.1 m/s	Peak tibial acceleration (g) via accelerometer
Sheerin et al. [31]	65 M, 20 F	39.6 ± 9.0	176 ± 9	73.9 ± 11	Runners (45.0 ± 23.2)	NA	2.7 m/s	3.7 m/s	Peak tibial acceleration (g) via accelerometer
Lam et al. [48]	18 M, 0 F	25 ± 2.3	179 ± 4.6	74.4 ± 6.5	Collegiate basketball players (NS)	NA	3.0 m/s	6.0 m/s	Tibial shock (g) via an accelerometer
Edwards et al. [19]	10 M, 0 F	24.9 ± 4.7	170 ± 10	70.1 ± 8.9	> Participated in general physical activity requiring running (NS)	NA	2.5 m/s	3.5 m/s	Peak principal strain estimated via finite element modelling of model tibia
Bonnaerens et al. [60]	30 M, 0 F	23 ± 1.9	181 ± 5.0	74 ± 6.0	Active in sport (NS)	NA	2.10 m/s	3.20 m/s	Peak tibial acceleration (g) via accelerometer
Greenhalgh et al. [61]	9 M, 0 F	21 ± 1.69	175.75 ± 6.56	78.13 ± 12.11	Elite university-level hockey players (NS)	NA	3.3 m/s	5.0 m/s	Peak tibial acceleration (g) via an accelerometer
Mercer et al. [62]	8 M, 0 F	25 ± 4.6	179 ± 6	80 ± 8.9	Physically active (NS)	NA	3.2 m/s	5.7 m/s	Peak tibial acceleration (g) via accelerometer
Mercer et al. [62]	8 M, 0 F	25 ± 4.6	179 ± 6	80 ± 8.9	Physically active (NS)	NA	3.2 m/s	6.4 m/s	Peak tibial acceleration (g) via accelerometer
Edwards et al. [19]	10 M, 0 F	24.9 ± 4.7	170 ± 10	70.1 ± 8.9	> Participated in general physical activity requiring running (NS)	NA	2.5 m/s	4.5 m/s	Peak principal strain estimated via finite element modelling of model tibia
Sinclair et al. [46]	10 M, 0 F	20.42 ± 3.55	178.75 ± 5.81	76.58 ± 6.52	Recreational runners (≥ 35)	NA	3.5 m/s	5.0 m/s	Peak tibial acceleration (g) via accelerometer

*NS* not specified, *NA* not applicable, *M* males, *F* females

**Table 6:** Participant and intervention characteristics of studies included in the surface category

Study	*N*	Age (years)	Height (cm)	Mass (kg)	Population activity (km run per week)	Subcategory (if applicable)	Control condition	Intervention condition	Tibial load measure
Fu et al. [56]	13 M, 0 F	23.7 ± 1.2	173.7 ± 5.7	65.7 ± 5.2	Recreational runners (20.4 ± 5.2)	Grass	Concrete	Grass	Peak positive tibial acceleration (g) via an accelerometer placed on the tibial tuberosity
Milner et al. [58]	9 M, 10 F	31 ± 6	170 ± 8	68.6 ± 11.6	Healthy runners (≥ 16.1)	Grass	Concrete	Grass	Peak tibial acceleration (g) via accelerometer
Fu et al. [56]	13 M, 0 F	23.7 ± 1.2	173.7 ± 5.7	65.7 ± 5.2	Recreational runners (20.4 ± 5.2)	Padded treadmill	Standard treadmill	Treadmill lined with EVA foam	Peak positive tibial acceleration (g) via an accelerometer placed on the tibial tuberosity
Milner et al. [58]	9 M, 10 F	31 ± 6	170 ± 8	68.6 ± 11.6	Healthy runners (≥ 16.1)	Synthetic surface	Concrete	Synthetic laboratory runway	Peak tibial acceleration (g) via accelerometer
Fu et al. [56]	13 M, 0 F	23.7 ± 1.2	173.7 ± 5.7	65.7 ± 5.2	Recreational runners (20.4 ± 5.2)	Synthetic surface	Concrete	Synthetic athletics track	Peak positive tibial acceleration (g) via an accelerometer placed on the tibial tuberosity
Greenhalgh et al. [61]	9 M, 0 F	21 ± 1.69	175.75 ± 6.56	78.13 ± 12.11	Elite university-level hockey players (NS)	Synthetic surface	Concrete	Synthetic hockey pitch	Peak tibial acceleration (g) via an accelerometer
Boey et al. [59]	18 M, 17 F	23.3 ± 3.33	175.77 ± 8.71	64.74 ± 7.69	Untrained (< 2), recreational (18 ± 7) and well-trained runners (74 ± 19)	Synthetic surface	Concrete	Synthetic athletics track	Positive vertical acceleration peaks (g) via accelerometer
Boey et al. [59]	18 M, 17 F	23.3 ± 3.33	175.77 ± 8.71	64.74 ± 7.69	Untrained (< 2), recreational (18 ± 7) and well-trained runners (74 ± 19)	Woodchips	Concrete	Woodchip trail	Positive vertical acceleration peaks (g) via accelerometer

*NS* not specified, *M* males, *F* females

**Table 7:** Participant and intervention characteristics of studies included in the technique category

Study	*N*	Age (years)	Height (cm)	Mass (kg)	Population activity (km run per week)	Subcategory (if applicable)	Control condition	Intervention condition	Tibial load measure
Huang et al. [17]	19 M, 0 F	21.74 ± 2.64	178.84 ± 5.43	68.48 ± 6.28	Recreational runners (17.92 ± 10.15)	Increased anterior trunk lean	Preferred gait	10-degree increase in preferred anterior trunk	Peak tibial acceleration (g) via an accelerometer
Huang et al. [17]	19 M, 0 F	21.74 ± 2.64	178.84 ± 5.43	68.48 ± 6.28	Recreational runners (17.92 ± 10.15)	Increased cadence	Preferred gait	10% increase in preferred cadence	Peak tibial acceleration (g) via an accelerometer
Yong et al. [18]	6 M, 11 F	32.1 ± 9.8	168 ± 11	64.9 ± 12.5	Recreational runners (33.5 km ± 17.5)	Increased cadence	Preferred gait	10% increase in cadence	Peak tibial acceleration (g) via an accelerometer
Olin and Gutierrez [45]	6 M, 12 F	32.2 ± 7.9	172 ± 10	68.6 ± 11.9	Recreational runners (20.9 ± 6.0)	Foot strike	Rearfoot strike pattern while barefoot	Forefoot strike pattern while barefoot	Peak tibial shock (g) via accelerometer
Lucas-Cuevas et al. [44]	22 NS	28.4 ± 5.8	173.6 ± 5.9	68.5 ± 6.6	Recreational runners (38.6 ± 15.4)	Foot strike	Rearfoot strike pattern while barefoot	Forefoot strike pattern while barefoot	Peak tibial acceleration (g) via accelerometer
Huang et al. [17]	19 M, 0 F	21.74 ± 2.64	178.84 ± 5.43	68.48 ± 6.28	Recreational runners (17.92 ± 10.15)	Foot strike	Rearfoot strike pattern	Forefoot strike pattern	Peak tibial acceleration (g) via an accelerometer
Yong et al. [18]	6 M, 11 F	32.1 ± 9.8	168 ± 11	64.9 ± 12.5	Recreational runners (33.5 km ± 17.5)	Foot strike	Rearfoot strike pattern	Forefoot strike pattern	Peak tibial acceleration (g) via an accelerometer
Laughton et al. [26]	15 NS	22.46 ± 4	169.75 ± 6.07	66.41 ± 8.58	Runners (NS)	Foot strike	Rearfoot strike pattern	Forefoot strike pattern	Peak positive tibial acceleration (g) via an accelerometer
Crowell et al. [64]	0 M, 5 F	26 ± 2	164 ± 6	53.9 ± 5.4	Recreational runners (≥ 32)	Real-time biofeedback	Warm-up, running with no prior visual feedback of peak positive tibial accelerations	Running with visual feedback, with the aim to keep peak tibial acceleration below a threshold	Peak positive tibial acceleration (g) via an accelerometer
Crowell et al. [64]	0 M, 5 F	26 ± 2	164 ± 6	53.9 ± 5.4	Recreational runners (≥ 32)	Real-time biofeedback	Warm-up, running with no prior visual feedback of peak positive tibial accelerations	Running with no feedback post visual feedback, with the aim to retain low tibial accelerations	Peak positive tibial acceleration (g) via an accelerometer
Wood and Kipp [65]	3 M, 6 F	20 ± 15	170.2 ± 8.7	59.1 ± 8.2	Recreational runners (≥ 32.2)	Real-time biofeedback	Warm-up, running with no prior audio feedback of peak positive tibial accelerations	Running with audio feedback, with the aim to keep peak tibial acceleration below a threshold	Peak positive tibial acceleration (g) via an accelerometer
Wood and Kipp [65]	3 M, 6 F	20 ± 15	170.2 ± 8.7	59.1 ± 8.2	Recreational runners (≥ 32.2)	Real-time biofeedback	Warm-up, running with no prior audio feedback of peak positive tibial accelerations	Running no feedback post audio feedback, with aim to retain low tibial accelerations	Peak positive tibial acceleration (g) via an accelerometer
Bonnaerens et al. [60]	30 M, 0 F	23 ± 1.9	181 ± 5.0	74 ± 6.0	Active in sport (NS)	Grounded running	Normal aerial running	Grounded running, no flight phase	Peak tibial acceleration (g) via an accelerometer
Edwards et al. [21]	10 M, 0 F	22.2 ± 3.2	180 ± 10	69.2 ± 6.5	Experienced runners (NS)	Decreased stride length	Preferred gait	10% decrease in preferred stride length	Peak resultant tibial contact forces (bw) estimated via finite element modelling of model tibia
Derrick et al. [66]	10 M, 0 F	27 ± 5	179 ± 5	75.5 ± 12.2	NS (NS)	Decreased stride length	Preferred gait	10% decrease in preferred stride length	Peak tibial acceleration (g) via an accelerometer
Derrick et al. [66]	10 M, 0 F	27 ± 5	179 ± 5	75.5 ± 12.2	NS (NS)	Decreased stride length	Preferred gait	20% decrease in preferred stride length	Peak tibial acceleration (g) via an accelerometer
Derrick et al. [66]	10 M, 0 F	27 ± 5	179 ± 5	75.5 ± 12.2	NS (NS)	Increased stride length	Preferred gait	10% increase in preferred stride length	Peak tibial acceleration (g) via an accelerometer
Derrick et al. [66]	10 M, 0 F	27 ± 5	179 ± 5	75.5 ± 12.2	NS (NS)	Increased stride length	Preferred gait	20% increase in preferred stride length	Peak tibial acceleration (g) via an accelerometer
Meardon and Derrick [16]	8 M, 7 F	23.7 ± 5.4	170 ± 8	70.3 ± 9.2	Experienced runners (≥ 16.1)	Decreased step width	Preferred gait	Narrow step width	Peak normal and shear stress calculated via musculoskeletal modelling
Meardon and Derrick [16]	8 M, 7 F	23.7 ± 5.4	170 ± 8	70.3 ± 9.2	Experienced runners (≥ 16.1)	Increased step width	Preferred gait	Wider step width	Peak normal and shear stress calculated via musculoskeletal modelling

*NS* not specified, *M* males, *F* females

### Risk of Bias

**Fig. 2:**
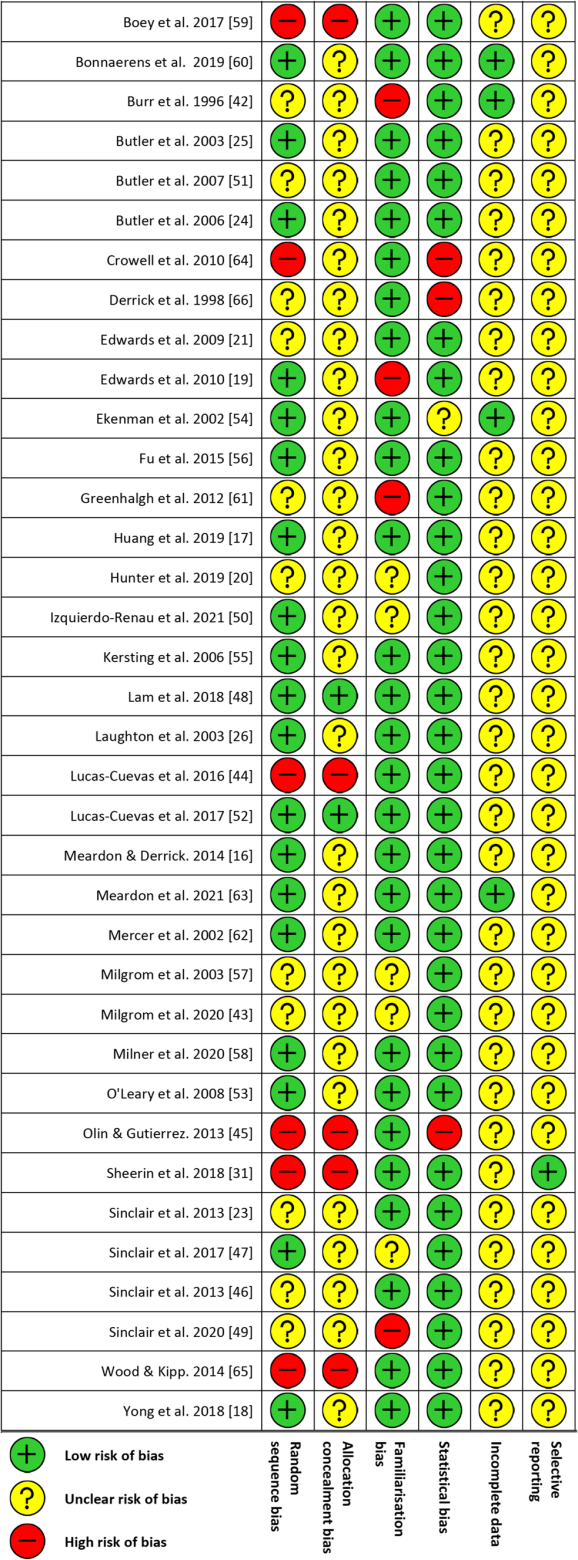
Risk-of-bias assessment for all included studies

Risk-of-bias assessments are reported in Fig. 2. Six studies reported a high risk of random sequence bias, five studies reported high risk of allocation concealment bias, four studies reported high risk of familiarisation bias and three studies reported high risk of statistical bias. None of the included studies were found to have a high risk of bias for the selective reporting or incomplete data criterion. No studies were removed due to risk-of-bias outcomes.

### Quality Assessment

All studies were of crossover design, with all categories except speed having fewer than 100 participants; hence, GRADE identified all pooled variables as having a very low certainty of evidence (see Table 8).

**Table 8:** Summary of meta-analysis findings and certainty of evidence. Only categories that could be pooled have been included

Intervention	Summary of findings				Quality of pooled data (GRADE)			
	S	K	N	Effect (95% CI)	Imprecision	Inconsistency	Risk of bias	Overall certainty
Barefoot	4	4	62	1.16 (0.50, 1.82)	− 1	− 1	− 1	Very low
Footwear								
Increased cushioning	1	2	36	− 0.30 (− 0.75, 0.16)	− 1	0	0	Very low
Minimalist shoe (habitual wearers)	2	2	32	0.34 (− 1.33, 2.00)	− 1	− 1	− 1	Very low
Minimalist shoes (non-habitual wearers)	3	3	32	0.89 (0.40, 1.39)	− 1	0	0	Very low
Motion control shoe	2	3	52	0.46 (0.07, 0.84)	− 1	0	0	Very low
Orthotics, insoles and taping								
Cushioned insoles	2	3	93	− 0.03 (− 0.32, 0.25)	− 1	0	0	Very low
Rigid orthotics	2	2	30	− 0.10 (− 0.59, 0.40)	− 1	0	0	Very low
Soft orthotics	2	2	24	0.03 (− 0.51, 0.58)	− 1	0	0	Very low
Taping and bracing	1	3	30	− 0.05 (− 0.54, 0.43)	− 1	0	0	Very low
Overground vs. treadmill	3	3	36	− 0.83 (− 1.53, − 0.12)	− 1	0	0	Very low
Speed	10	21	583	0.87 (0.61, 1.13)	0	− 1	0	Very low
Surface								
Grass	2	2	32	− 0.21 (− 0.69, 0.27)	− 1	0	0	Very low
Synthetic surface	4	4	76	− 0.45 (− 0.98, 0.09)	− 1	− 1	− 1	Very low
Technique								
Increased cadence	2	2	36	− 0.25 (− 0.88, 0.37)	− 1	0	0	Very low
Forefoot strike	5	5	91	− 0.84 (− 2.41, 0.72)	− 1	− 1	0	Very low
Real-time biofeedback	2	4	28	− 0.93 (− 1.46, − 0.41)	− 1	0	− 1	Very low
Increased stride length	1	2	20	0.86 (0.18, 1.55)	− 1	0	− 1	Very low
Decreased stride length	2	3	30	− 0.30 (− 0.79, 0.19)	− 1	0	− 1	Very low

*S* number of studies, *K* number of included outcomes, *N* total number of participants, *CI* confidence interval, *GRADE* Grading of Recommendations Assessment, Development and Evaluation system

### General Findings

#### Barefoot

**Fig. 3:**
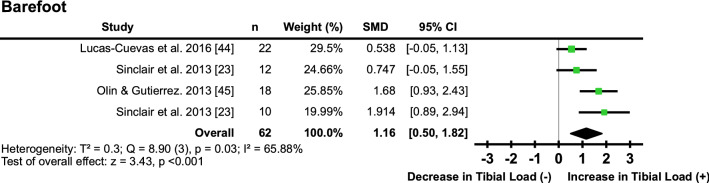
Pooled effects of tibial loads during barefoot running compared with shod. *SMD* standardised mean difference, *CI* confidence interval, *T*^2^ Tau^2^, *Q* Chi^2^, *I*^2^ Higgins *I*^2^ statistic

Four studies [23, 44–46] examined the effect of barefoot running compared with shod on tibial loading. Barefoot running ‘moderately’ increased tibial load measurements with medium heterogeneity (*I*^2^ = 65.88%, SMD 1.16; 95% CI 0.50, 1.82) (Fig. 3; Table 1).

#### Footwear

**Fig. 4:**
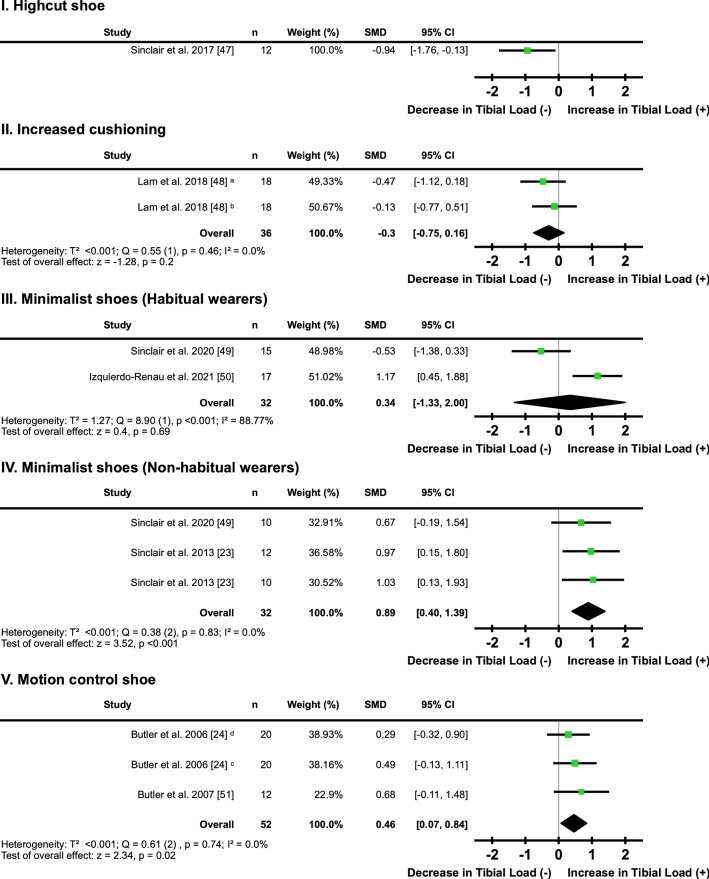
Individual and pooled effects of tibial loads when running in a **I.** high-cut shoe; **II.** shoe with increased cushioning; **III.** minimalist shoe (habitual wearers); **IV.** minimalist shoe (non-habitual wearers); and **V.** motion control shoe compared with a conventional running shoe. Where two or more interventions from the same study were included in a subcategory, symbols were used to distinguish the difference. ^a^Medium cushioning; ^b^high cushioning; ^c^high-arched participant pool; ^d^low-arched participant pool. *SMD* standardised mean difference, *CI* confidence interval, *T*^2^ Tau^2^, *Q* Chi^2^, *I*^2^ Higgins *I*^2^ statistic

One study [47] examined the effect of high-cut shoes on tibial loading and found a ‘moderate’ decrease in tibial loading (*I*^2^ = not applicable, SMD − 0.94; 95% CI − 1.76, − 0.13) (Fig. 4a; Table 2). One study [48] examined the effects of two different levels of shoe cushioning on tibial loading compared with a conventional running shoe, with no clear directional effect (*I*^2^ = 0%, SMD − 0.30; 95% CI − 0.75, 0.16) (Fig. 4b, Table 2). Two studies [49, 50] comparing minimalist shoe use in habitual wearers with the use of a conventional running shoe found no clear directional effect on tibial loading (*I*^2^ = 88.77%, SMD 0.34; 95% CI − 1.33, 2.00) (Fig. 4c, Table 2). Three studies [23, 46, 49] comparing minimalist shoe use in non-habitual wearers with use of a conventional running shoe found a ‘moderate’ increase in tibial loading (*I*^2^ = 0%, SMD 0.89; 95% CI 0.40, 1.39) (Fig. 4d, Table 2). Two studies [24, 51] with three different cohorts examined the effect of a motion control shoe, compared with a conventional running shoe, on tibial loading. Motion control shoes resulted in a ‘small’ increase in tibial loading (*I*^2^ = 0%, SMD 0.46; 95% CI 0.07, 0.84) (Fig. 4e, Table 2).

#### Orthotics, Insoles and Taping

**Fig. 5:**
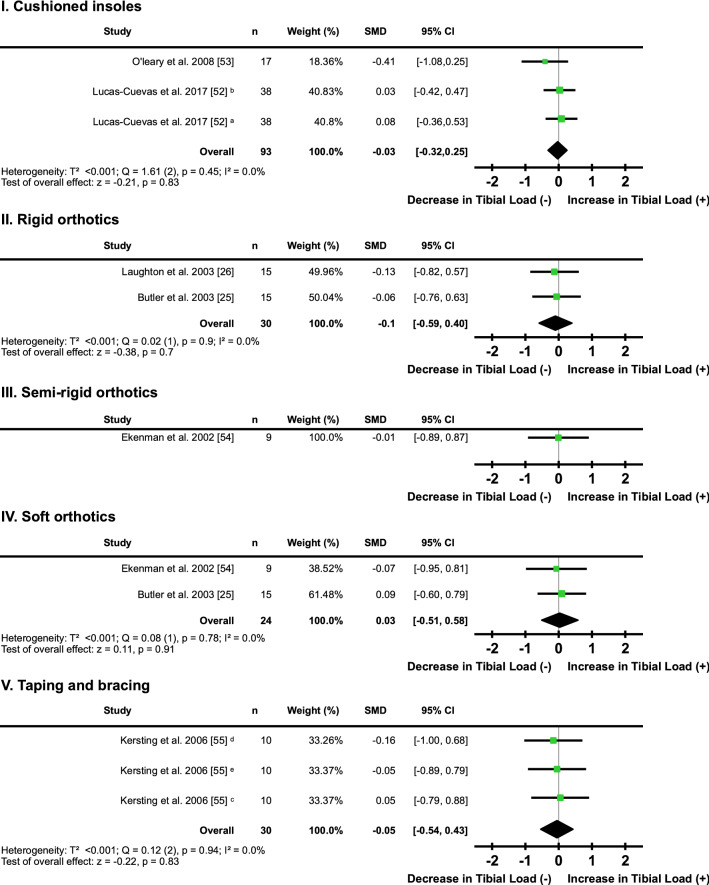
Individual and pooled effects of tibial load when running in a conventional shoe with **I.** cushioned insoles; **II.** rigid orthotics; **III.** semi-rigid orthotics; **IV.** soft orthotics; and **V.** taping and bracing compared with a conventional running shoe without intervention. Where two or more interventions from the same study were included in a subcategory, symbols were used to distinguish the difference. ^a^Prefabricated insoles; ^b^custom insoles; ^c^tape; ^d^brace/cast; ^e^tape with brace/cast. *SMD* standardised mean difference, *CI* confidence interval, *T*^2^ Tau^2^, *Q* Chi^2^, *I*^2^ Higgins *I*^2^ statistic

Two studies [52, 53] found that three different cushioned insoles did not have a clear directional effect on tibial loading (*I*^2^ = 0%, SMD − 0.03; 95% CI − 0.32, 0.25) (Fig. 5a, Table 3). Two studies [25, 26] found rigid orthotics did not have a clear directional effect on tibial loading (*I*^2^ = 0%, SMD − 0.10; 95% CI − 0.59, 0.40) (Fig. 5b, Table 3). One study [54] found semi-rigid orthotics did not have a clear directional effect on tibial loading (*I*^2^ = not applicable, SMD − 0.01; 95% CI − 0.89, 0.87) (Fig. 5c, Table 3). Two studies [25, 54] found soft orthotics did not have a clear directional effect on tibial loading (*I*^2^ = 0%, SMD − 0.03; 95% CI − 0.51, 0.58) (Fig. 5d, Table 3). One study [55] reported that three different taping and bracing techniques did not have a clear directional effect on tibial loading (*I*^2^ = 0%, SMD − 0.05; 95% CI − 0.54, 0.43) (Fig. 5e, Table 3).

#### Overground Versus Treadmill

**Fig. 6:**
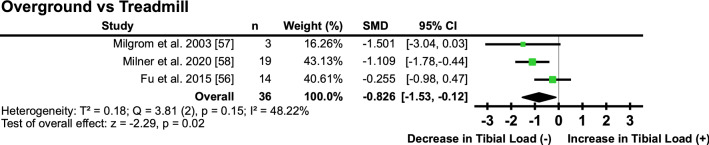
Pooled effects of tibial loads during overground running compared with treadmill. *SMD* standardised mean difference, *CI* confidence interval, *T*^2^ Tau^2^, *Q* Chi^2^, *I*^2^ Higgins *I*^2^ statistic

Three studies [56–58] reported that running on a treadmill, compared with running overground, resulted in a ‘moderate’ decrease in tibial loading (SMD − 0.83; 95% CI − 1.53, − 0.12) (Fig. 6, Table 4), with a ‘small’ amount of heterogeneity detected between interventions (*I*^2^ = 48.22%) (Fig. 6).

#### Speed

**Fig. 7:**
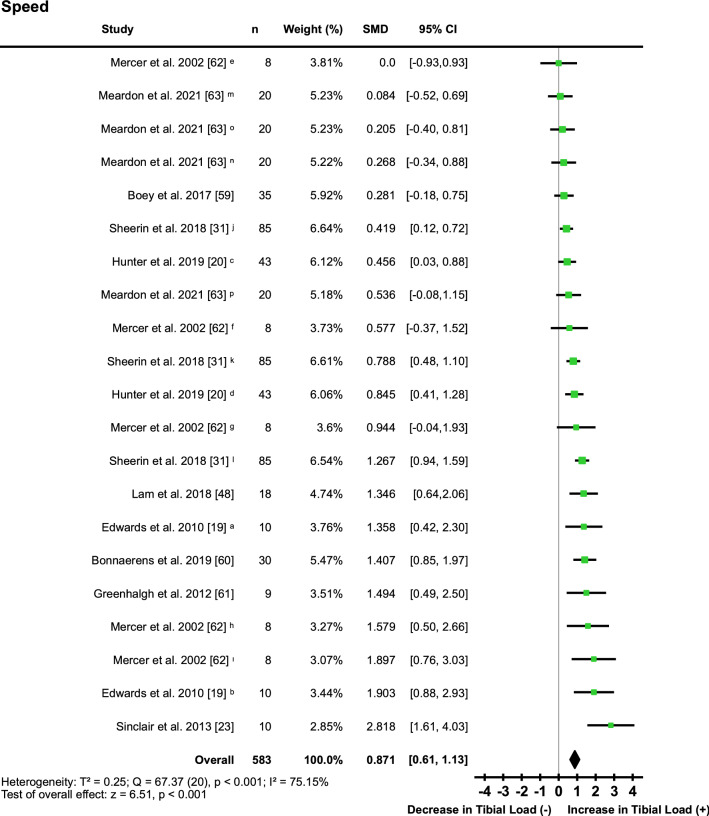
Pooled effects of tibial load when running with increased speed. Where two or more interventions from the same study were included in the speed category, symbols were used to distinguish the difference. ^a^2.5 m/s vs. 3.5 m/s; ^b^2.5 m/s vs. 4.5 m/s; ^c^2.7 m/s vs. 3.27 m/s; ^d^2.7 m/s vs. 4.08 m/s; ^e^3.2 m/s vs. 3.8 m/s; ^f^3.2 m/s vs. 4.5 m/s; ^g^3.2 m/s vs. 5.1 m/s; ^h^3.2 m/s vs. 5.7 m/s; ^i^3.2 m/s vs. 6.4 m/s; ^j^2.7 m/s vs. 3.0 m/s; ^k^2.7 m/s vs. 3.3 m/s; ^l^2.7 m/s vs. 3.7 m/s; ^m^90% preferred vs. preferred speed (female population); ^n^90% preferred vs. preferred speed (female population); ^o^90% preferred vs. preferred speed (male population); ^p^90% preferred vs. 110% preferred speed (male population). *SMD* standardised mean difference, *CI* confidence interval, *T*^2^ Tau^2^, *Q* Chi^2^, *I*^2^ Higgins *I*^2^ statistic

Ten studies [19, 20, 31, 46, 48, 59–63] reported 21 changes in speed. Increased speed resulted in a ‘moderate’ increase in tibial loading (SMD 0.87; 95% CI 0.61, 1.13) (Fig. 7, Table 5). A statistically significantly moderate to high level of heterogeneity was detected between studies (*I*^2^ = 75.15%) (Fig. 7).

#### Surface

**Fig. 8:**
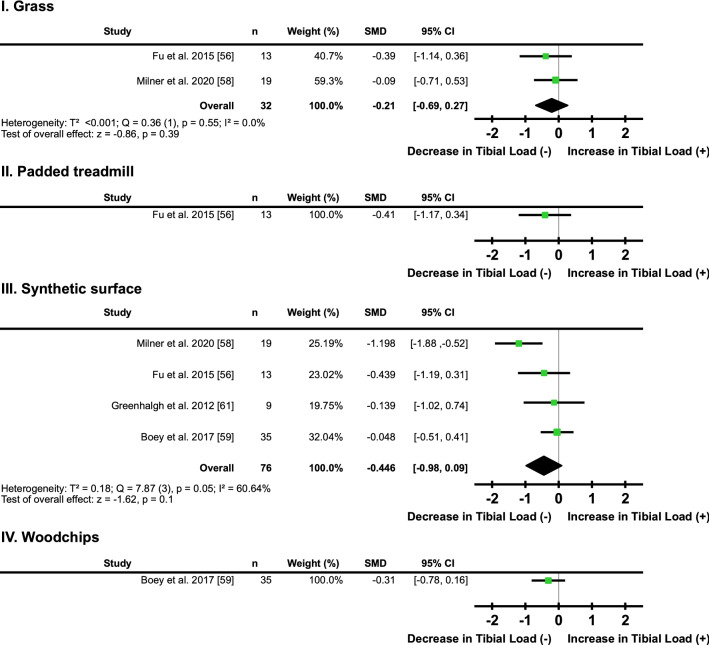
Individual and pooled effects of tibial loads when running on **I.** grass, **II.** a padded treadmill, **III.** a synthetic surface; and **IV.** a woodchip surface compared to a stiffer less compliant surface. *SMD*-Standardised mean difference; *95% CI* confidence interval, *T*^2^ Tau^2^, *Q* Chi^2^, *I*^2^ Higgins *I*^2^ statistic

Two studies [56, 58] found that changing from concrete to grass did not have a clear directional effect on tibial loading (*I*^2^ = 0%, SMD − 0.21; 95% CI − 0.69, 0.27) (Fig. 8a, Table 6). One study [56] found that changing from a normal to a padded treadmill had no clear directional effect on tibial loading (*I*^2^ = not applicable, SMD − 0.41; 95% CI − 1.17, 0.34) (Fig. 8b, Table 6). Four studies [56, 58, 59, 61] found that changing from concrete to a synthetic surface did not have a clear directional effect on tibial loading (SMD − 0.45; 95% CI − 0.98, 0.09) (Fig. 8c, Table 6), with a ‘moderate’ level of heterogeneity detected between these studies (*I*^2^ = 60.64%) (Fig. 8c). One study [59] found that changing from a concrete to a woodchip track did not have a clear directional effect on tibial loading (*I*^2^ = not applicable, SMD − 0.31; 95% CI − 0.78, 0.16) (Fig. 8d, Table 6).

#### Technique

**Fig. 9:**
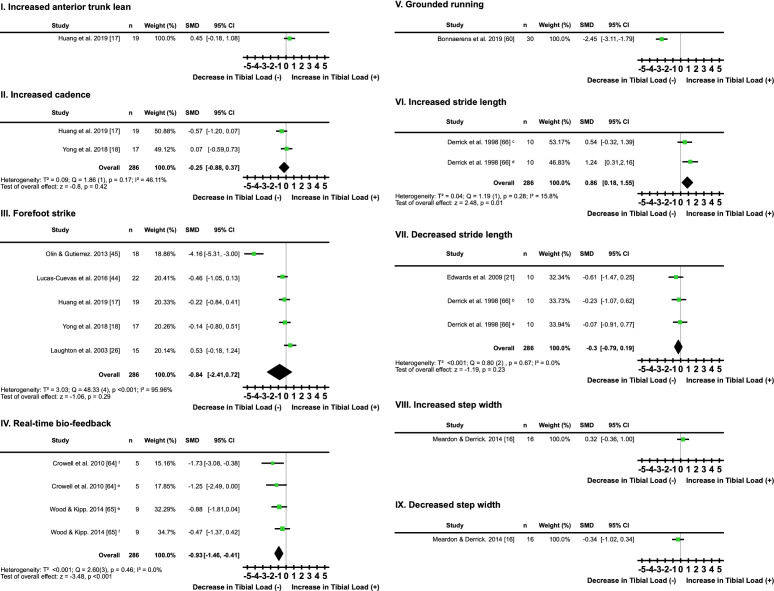
Individual and pooled effects of tibial load of interventions that modified running technique by **I.** increasing anterior trunk lean; **II.** increasing cadence; **III.** changing runners to a forefoot strike; **IV.** using real-time biofeedback; **V.** using grounded running; **VI.** increasing stride length; **VIII.** decreasing stride length; **IX.** increasing step width; and **i** decreasing step width. Where two or more interventions from the same study were included in a subcategory, symbols were used to distinguish the difference. ^a^− 20% of preferred stride length; ^b^− 10% of preferred stride length; ^c^+ 10% of preferred stride length; ^d^+ 20% of preferred stride length; ^e^visual feedback period; ^f^post-visual feedback period. *SMD* standardised mean difference, *CI* confidence interval, *T*^2^ Tau^2^, *Q* Chi^2^, *I*^2^ Higgins *I*^2^ statistic

One study [17] found that increasing anterior trunk lean by 10° had no clear directional effect on tibial loading (*I*^2^ = not applicable, SMD 0.45; 95% CI − 0.18, 1.08) (Fig. 9a, Table 7). Two studies [17, 18] found that increasing cadence by 10% had no clear directional effect on tibial loading (*I*^2^ = 46.11%, SMD − 0.25; 95% CI − 0.88, 0.37) (Fig. 9b, Table 7). Five studies [17, 18, 26, 44, 45] found that changing from a rearfoot to a forefoot strike pattern did not have a clear directional effect on tibial loading (SMD − 0.84; 95% CI − 2.41, 0.72) (Fig. 9c, Table 7). There was also an ‘extremely high’ level of heterogeneity between studies on foot strike pattern (*I*^2^ = 95.96%). Two studies [64, 65] reporting four biofeedback variables found that real-time biofeedback decreased tibial loading ‘moderately’ (*I*^2^ = 0%, SMD − 0.93; 95% CI − 1.46, − 0.41) (Fig. 9d, Table 7). One study [60] found that transitioning to grounded running resulted in a ‘very large’ decrease in tibial loading (*I*^2^ = not applicable, SMD − 2.45; 95% CI –3.11, − 1.79) (Fig. 9e, Table 7). One study [66] found that increasing stride length causes a ‘moderate’ increase in tibial loading (*I*^2^ = 15.80%, SMD 0.86; 95% CI 0.18, 1.55) (Fig. 9f, Table 7), while two studies [21, 66] found that decreasing stride length had no clear directional effect on tibial loading (*I*^2^ = 0%, SMD − 0.30; 95% CI − 0.79, 0.19) (Fig. 9g, Table 7). One study [16] found that increasing step width had no clear effect on tibial loading (*I*^2^ = not applicable, SMD 0.32; 95% CI − 0.36, 1.00) (Fig. 9h, Table 7). Similarly, decreasing step width also had no clear directional effect (*I*^2^ = not applicable, SMD − 0.34; 95% CI − 1.02, 0.34) (Fig. 9i, Table 7).

## Discussion

The magnitude of tibial loading during running is an important risk factor for tibial stress injuries [35, 67]. A wide array of interventions have been adopted to modify tibial loads during running [16, 20, 21, 65]. This review found the greatest increase in tibial loading when individuals ran barefoot compared with shod (SMD 3.43). Increased tibial load was also found for non-habitual users running in a minimalist shoe (SMD 0.89), increased running speed (SMD 0.87), increased stride length (SMD 0.86) and motion control shoe use (SMD 0.46). Tibial loading decreased with targeted biofeedback (SMD − 0.93) and when running on a treadmill versus overground (SMD − 0.83). These findings can be useful to prescribe interventions to potentially reduce tibial loading during training or when returning from tibial stress injury.

This review found that increases in running speed moderately increase tibial loading. A significantly moderate to high level of heterogeneity was detected between studies (*I*^2^ = 74.80%), likely explained by the variability in speeds used. While all studies increased speed, the magnitude of increase and baseline speeds varied among studies, which created considerable variability in individual effect sizes. Irrespective of the variability, there was a consistent effect for elevated tibial loading with an increase in running speed. Due to high heterogeneity, the magnitude of the overall effect size should be interpreted with caution. Furthermore, tibial stress injuries are multifactorial and high-speed running speeds combined with other factors such as high training volume or limited rest could increase the risk of injury.

This review also found that as individuals increase above preferred stride length, a moderate increase in tibial loads occurred. An increase in stride length has been noted to increase ground reaction forces during running [68], and these increases may result in larger braking forces being absorbed by the tibia [62, 66]. However, this review found a decrease in preferred stride length had no clear directional effect on tibial loading. This outcome is both supported and contradicted by the kinematics and kinetics of altered stride length. One study found that hip, knee, and ankle joint moments did not change when stride length was decreased in a shod condition, yet vertical ground reaction forces decreased [68]. Another study found decreased stride length (resulting in increased stride frequency) can reduce joint loads, vertical impact peaks, vertical instantaneous and average loading rate [69]. As previously noted, reaction forces alone are not well correlated with bone strain measures and may only have a small contribution to tibial loads [33]. More research may be warranted to gain better insight into the effects decreasing stride length has on tibial loading, and the associated mechanisms driving any potential change.

No clear directional effect was found on tibial loading when runners increased cadence and changed to a forefoot strike. There were limited studies examining altered cadence, with the two studies [17, 18] included having opposite directional effects (SMD − 0.57 vs. SMD 0.07). A few differences that could have potentially affected the outcomes were the difference in participant sex (male only vs. males and females), average age (21.7 ± 2.6 vs. 32.1 ± 9.8), the difference in the weekly running mileage of participants (17.1 ± 10.1 vs. 33.5 ± 17.5) and the total number of outcome variables tested in a session (11 vs. 3) [17, 18]. Other aspects of the studies were very similar, including the placement of tibial accelerometers. The mixed evidence presented suggests further investigation into the effects of cadence on tibial loading is warranted. Variable outcomes of included studies [17, 18, 26, 44, 45] also played a role in the inconclusive results of foot strike interventions. This was reflected in the high heterogeneity reported between the included studies (*I*^2^ = 95.96%). A potential reason for this high variance was that these studies included a mix of normal shod [17, 18, 26] and barefoot running [44, 45]. It is possible that shifting to a forefoot strike under these different footwear conditions has a variable effect on any changes in tibial loads.

Our results indicated a moderate decrease in tibial loading when audio or visual biofeedback was used. The studies in this analysis used a sound or visual cue to keep tibial acceleration peaks under a set threshold. It is unclear if these reductions were caused by kinetics; one included study found participants with the greatest reduction in accelerations also showed reductions in ground reaction forces, and instantaneous and average loading rates [64]. Although these changes were observed, ground reaction forces are often only a small contributor to loads experienced by the bone [19, 70]. It is also unclear if the observed reductions occurred due to changes in running kinematics, as these were not reported in either study [64, 65]. The relatively simplistic nature of these feedback processes suggests biofeedback could be a useful approach for reducing tibial loading in the field (e.g., including sound cues in a runner’s headphones). There is also the potential that biofeedback has long-term effects on tibial load measures. Decreases in peak tibial accelerations have been observed 1 month after completing gait retraining using visual biofeedback [71]. However, further studies with longer-term follow-ups are required to better understand the retention of gait retraining interventions in real-world scenarios.

Barefoot compared with shod running moderately increased tibial load measures. The studies [23, 44–46] within this analysis all used novice barefoot runners and may therefore not reflect tibial load changes in habitual barefoot runners. During early exposure to barefoot running, habitually shod runners may not alter landing patterns from a heel strike to a mid/forefoot pattern [72]. Individuals who naturally run with a rear foot strike pattern may be prone to increased lower limb loading during the impact phase of running when transitioning to barefoot [73]. These results suggest that the introduction of barefoot running to those inexperienced with the concept could elevate tibial loads. Although no studies in this review included habitual barefoot runners, a similar notion was observed when considering the use of minimalist shoes. Our review found that minimalist shoes worn by those unaccustomed to this footwear moderately increased tibial loading. The same increase in tibial loading was not observed in habitual minimalist shoe users; however these results are likely influenced by survivor bias, where included subjects were unlikely to experience any negative effects of minimalist shoes, allowing them to become habitual users. Hence, these results may not broadly represent the entire running population. Where a runner plans to shift to barefoot running or running in a minimalist shoe, a gradual introduction may be required to not increase the risk of tibial stress injury. Furthermore, introducing barefoot running or minimalist shoes in those who are rehabilitating or at risk of a tibial stress injury should likely be avoided.

This review also found increases in tibial loads when a motion control shoe was adopted during running. The aim of a motion control shoe is to reduce rear foot eversion during running and walking [51]. A consequence of increased rearfoot eversion may be an altered load distribution within the lower extremity, predisposing individuals to a tibial stress injury [74]. Elevated rear foot eversion while running has been cited as a potential risk factor for tibial stress injuries [74, 75]. Although the included studies indicated that rearfoot eversion was reduced with motion control shoes, the meta-analysis still found small increases in tibial loading. Despite the motion control shoes targeting a mechanism thought to be linked to tibial stress injury risk (i.e., rearfoot eversion), this did not translate to a reduction in measured tibial loads. The evidence from this review suggests that motion control shoes may not reduce tibial loads during running.

Only one study was identified that examined high- versus low-cut shoes [47]. The study indicated that there was a ‘moderate’ decrease in tibial loading when wearing a high-compared with low-cut football cleat (SMD − 0.94) [47]. High-cut shoes may be beneficial in reducing tibial loads when worn during running activities; however these assumptions are currently only applicable to football cleats. This may be beneficial to those who regularly wear football cleats, but the application of this research is limited and cannot be applied to the majority who are at risk of tibial stress injuries (i.e., distance runners). Further research is warranted in other types of high-cut footwear and is necessary before any conclusions can be made regarding the effect these may have on tibial loading in other running populations.

Cushioned shoes and insoles were found to not have a clear directional effect on tibial loading during running. Individuals adapt their running kinematics to match the stiffness of shoes and surfaces [76, 77]. A runner increases leg stiffness, particularly at the ankle joint, when running in softer shoes to maintain large enough ground reaction forces for running [76]. Increases in leg stiffness may increase the loads that are transmitted through the musculoskeletal system [77]. The above-mentioned adaptations may be why minimal changes were observed when comparing normal shoes with softer, more compliant shoes and insoles. This review also found that rigid and soft orthotics had no clear directional effect on tibial loading. Semi-rigid orthotics were also included in the study; however meta-analysis was not possible due to only one study examining this intervention. Our review also found no clear directional effect of ankle taping or bracing on tibial loading when used during running. Minimal effects on tibial load were likely observed due to a lack of change in foot and ankle biomechanics with these interventions [25, 26].

No clear directional effect was found when individuals changed from a concrete surface to a grass surface, and no clear directional effect was found when running on a synthetic surface compared with a concrete surface. Lastly, only one study looked at running on a padded treadmill versus a conventional treadmill, and only one study compared running on concrete and running on a woodchip surface; therefore, a meta-analysis was not performed for either of these interventions. The overarching aim of all these interventions is to produce a more cushioned, compliant running surface. As discussed earlier, individuals adapt their running kinematics to match the stiffness of shoes [76, 77], with these adaptations also extending to when running on softer surfaces; this could again explain why no changes in tibial stress measures were observed when running on softer surfaces. However, this review found a moderate decrease in tibial loading when individuals ran on a treadmill versus overground surface (e.g., concrete). The difference in stiffness between overground and treadmill surfaces is a theory for the changes in tibial load [78]; however, our results comparing surfaces suggest that this may not be the predominant reason for a reduction in tibial load during treadmill running. Treadmill running has been shown to reduce vertical displacement [78]. The reduced vertical displacement decreases the amount of vertical acceleration, which in turn decreases the vertical forces during running [78]. Individuals running on a treadmill may also reduce braking and propulsion forces compared with when running overground [78]. It is proposed that the altered loading conditions experienced when running on a treadmill versus overground are the more likely mechanisms responsible for the decreased tibial loading observed. There is evidence to suggest treadmill running may elevate tibial loading due to greater ankle joint moments and planter flexor muscle forces [78]. However, strain gauges attached directly to the tibia have shown decreases in both compressive and tensile loads of the tibia when running on a treadmill compared with overground [57]. Individuals in training periods of higher load or recovering from previous tibial stress injury could use treadmills to supplement overground training. This could potentially reduce overall or cumulative loads on the tibia and reduce the risk of injury/re-injury.

Several technique-based interventions could not be pooled for meta-analyses. These included increased and decreased step width, increased anterior trunk lean and grounded running. Modifications to step width and trunk lean had no directional effect on tibial loading, and therefore neither could be supported for modifying tibial load based on current evidence. Adopting a grounded running technique generated a ‘very large’ decrease in tibial accelerations (SMD − 2.45). This result is not particularly surprising, as the typically slower speeds and biomechanical alterations associated with grounded running reduce the biomechanical loading of the lower extremities [79]. Grounded running may therefore be a promising technique adaptation for reducing tibial loads, yet there is little understanding of the feasibility of gait-retraining techniques for promoting grounded running long-term [79].

### Limitations

This review included a vast number of tibial load variables; however the majority (75%) of the measures used to assume tibial load were accelerometry metrics from wearable sensors. Recent studies have indicated that wearable sensors may have the same poor correlations as ground reaction forces for the measurement of tibial loading, particularly at the bone level [33, 34]. However, the vertical average loading rate recent studies used to assume this poor correlation were found to be well correlated to estimates of bone force when running on level ground [33]. All studies included in this paper were run on a level ground. Reductions in accelerometry-based loads are also likely indicative of a ‘softer’ foot strike. There is recent evidence to suggest that ‘softer’ foot strike or running in a more flexed posture could increase muscle forces during running, causing an increase in bone loading that may be missed by accelerometry measures [80]. It has been identified that for every 1 g increase in peak positive acceleration, the likelihood of having a history of TSF increases by a factor of 1.361 [35]. The magnitude of peak tibial acceleration could also predict group membership (injured vs. uninjured) in 70% of cases [35]. Preliminary prospective findings have also indicated that individuals with tibial stress injury show 15% greater tibial acceleration than their uninjured counterparts [81]. This evidence suggests that acceleration measures may be a good predictor of tibial stress injury, and altering these measures could therefore be an important component of injury prevention. While the relationship between accelerometry and tibial stress needs further investigation, we acknowledge the limitations of tibial acceleration measures, particularly on inclined surfaces. As work in this area progresses, researchers should persist with higher-quality measures (e.g., bone-level stress estimates) to improve the understanding these interventions have on tibial loading. Further insights on the effect of muscle forces, tibial moments, tibial stress and strain are likely necessary to yield the greatest understanding of what interventions are the most effective.

The included studies contain a large amount of variance in the methodological processes used to understand the effects of the chosen interventions. These differences occur in the experimental procedures (running speed, placement of accelerometer, run time, etc.), measurement type used (accelerometry, estimation from musculoskeletal model, strain gauges, etc.) and the equipment used during these methods (variation in shoe brands etc.). A subjective decision to group interventions was made, despite certain variations in methodology, to provide an understanding of the broader overall effects of interventions. Due to this, some intervention categories examined have shown high heterogeneity, even when results are conclusive (e.g., speed).

There was also variation in the populations used across included studies, and all studies investigated healthy individuals. The physical activity/sport profiles of participants varied, with studies including team sport athletes, recreational runners, elite runners, and even sedentary populations. Due to the populations only being healthy individuals, it is unclear if these interventions would have the same effect on those who are presenting with pain, and current or existing tibial stress injuries. Caution should be exercised when applying the interventions to these populations.

This review only examined the immediate (i.e., within session) effects of interventions. Training variables such as weekly running volume, training frequency, and frequency of rest days are important to consider in the context of tibial stress injuries. Interpretation of results from this review should be considered in light of these other training variables that may affect tibial stress injuries.

Finally, the GRADE system assessed all pooled evidence as very low certainty. This was predominantly driven by the type of research (crossover trials) and the lack of participants. Considering this, caution may be necessary with the interpretation of these findings. There is a need for more robust, randomised trials with larger participant samples in the area of gait retraining interventions for modifying tibial loads during running.

## Conclusion

This review found that tibial loading increased when running barefoot, in motion control shoes, and when minimalist shoes were adopted by non-habitual users. Increased stride length and running speed were also found to increase tibial loading. These conditions may need to be avoided during training periods of high running volume or be avoided by runners recovering from a tibial stress injury. Running on a treadmill versus overground, as well as the use of biofeedback, can reduce tibial loading. These interventions could be adopted to reduce tibial load in healthy populations during training. These interventions may also be beneficial to individuals in rehabilitation from tibial stress injuries, but it is unknown if this population will respond the same or differently to the intervention. Caution should be exercised when interpreting for injured runners as the interventions included in this review may have not been evaluated in injured populations.

## Declarations

### Availability of data and material

The data analysed in this review can be found in the referenced published articles or from the corresponding author upon reasonable request.
